# Qualitative Analysis of Coaching With Care Partners of People With Cognitive Impairment

**DOI:** 10.1093/geroni/igab046.651

**Published:** 2021-12-17

**Authors:** Bianca Shieu, Chiao-Hsin Teng, Ya-Ning Chan, Youngmin Choi, Cass Dictus, Janelle Perez, Jing Wang, Victoria Bartoldus

**Affiliations:** 1 University of Pittsburgh/ School of Medicine, Pittsburgh, Pennsylvania, United States; 2 UNC Chapel Hill School of Nursing, Chapel Hill, North Carolina, United States; 3 UNC Chapel Hill, Chapel Hill, North Carolina, United States; 4 University of North Carolina at Chapel Hill School of Nursing, Chapel Hill, North Carolina, United States; 5 Fudan University, Shanghai, China; 6 University of North Carolina at Chapel Hill, Chapel Hill, North Carolina, United States

## Abstract

We apply the adaptive leadership framework for chronic Illness in a care partner–assisted intervention to improve oral hygiene of older adults with cognitive impairment. Care partners receive four coaching sessions which we recorded and transcribed verbatim. We will describe how our team of seven investigators codes the data using a priori codes from the framework. The data from 17 care-partners contributes 68 individual sessions for coding. We have two subgroups of 7 individuals with mild dementia (MD) and 10 with mild cognitive impairment (MCI). We will discuss the plan for multiple comparisons such as (a) longitudinal across 3 months of intervention, b) within MD and within MCI and c) between MD and MCI. To illustrate, we will discuss our approaches to reaching coding consensus and rigor and will present results of the within group analyses. Finally, we will discuss next steps and the end products we aim to achieve.

